# Brief intervention for the problems related to the use of alcohol: utilization among nurses

**DOI:** 10.1186/1940-0640-8-S1-A46

**Published:** 2013-09-04

**Authors:** Angélica Martins de Souza Gonçalves, Sonia Regina Zerbetto

**Affiliations:** 1 Department of Nursing, Federal University of São Carlos, São Carlos, São Paulo, Brazil

## 

A brief intervention is a simple and inexpensive resource that can be used by several professionals in different healthcare contexts. The present study aims to develop a reflection about the application of brief intervention and it utilization by nurses in a real-world work context. It recognized the importance of nurses’ performance on preventive actions, especially those acting under the auspices of primary care. The application of strategies for diagnosis and brief intervention is especially useful to reduce problems caused by alcohol use, because it can reach a large segment of the population before the individual has become alcohol dependent. Appropriate situations to consider the application of screening and brief intervention by nurses would be: during nursing consultation for adults users or the elderly, even if the consultation has been scheduled for another purpose; during activities aimed to draw people's awareness of a health topic (e.g., campaigns held in public places); during home visits; during groups (e.g., those for pregnant women or people with hypertension or diabetes). In this context, nurses should be taught the specific technologies and approaches to screening and brief intervention, but in a straightforward manner that allows its use by professionals without specialized formation.

